# Isolated cardiac hydatid cyst presented as myopericarditis: A case report

**DOI:** 10.34172/jcvtr.2020.13

**Published:** 2020-01-06

**Authors:** Farveh Vakilian, Akbar Kamali, Ali Azari, Hoorak Poorzand, Amir Kamali, Somayeh Vakili Ahrari Roodi

**Affiliations:** ^1^Cardiothoracic Research Center, Faculty of Medicine, Mashhad University of Medical Sciences, Mashhad, Iran; ^2^Department of Cardiology, Imam Reza Hospital, Faculty of Medicine, Mashhad University of Medical Sciences, Mashhad, Iran; ^3^Royan Institute for Stem Cell Biology and Technology, ACECR, Tehran, Iran; ^4^Department of Pathology, Imam Reza hospital, Mashhad University of Medical Sciences, Mashhad, Iran

**Keywords:** Cardiac Cyst, Interventricular Septum, Myopericarditis, Hydatid Disease, Surgery

## Abstract

Hydatidosis commonly affect the liver and lungs but in rare cases, it can involve heart tissue. A 42-year-old man from urban areas of Khorasan Razavi province, northeastern Iran, was referred to the cardiac clinic with palpitation, and atypical chest pain in 2018. Large pericardial effusion, reduced left ventricle systolic function was found. A cystic-like lesion was also seen in inter-ventricular septum in echocardiography and high-resolution computed tomography (HRCT). Urgent cardiac surgery was done because of echocardiographic evidence of tamponade. Although the serologic analysis was negative for hydatidosis, surgical excision of cyst and the subsequent histopathological findings revealed a hydatid cyst. In endemic areas, hydatidosis should be considered in differential diagnosis of any cystic-like lesions, even if the serological analysis is negative.

## Introduction

Hydatidosis (Echinococcosis) is one of the most important zoonotic disease and sporadic parasitic infection that cause significant health problem in developing countries.^1^ Human, as intermediate host of hydatidosis, can be infected by various subtypes including *Echinococcus granulosus*, *E. multilocularis*, and *E. vogeli*.^2^ Ingestion of unwashed or uncooked vegetables or foods which contaminated with the parasite eggs is the main route of acquiring the infection.^3^ This infection commonly involves liver (50%-70%) through releasing of the parasite embryo into the intestine tract and carried to the liver via the portal vein. However, sometimes this parasite can reach to the lungs (20%-30%), heart and other organs through bypass the liver via vena cava. Cardiac hydatidosis is a rare complication and its frequency is lesser than 2% (0.5%-2% of all cases).^4,5^

Here we have discussion about a case of isolated cardiac hydatid cyst causes myopericarditis which rarely seen in clinical presentation.

## Case Presentation

A 42-year-old man (construction worker) from urban area of Mashhad, Northeastern Iran, referred to the cardiology clinic of Imam Reza hospital with palpitation, since 2 weeks ago. He had occasionally atypical chest pain which was neither positional nor related to exertion.

**Figure 1:**
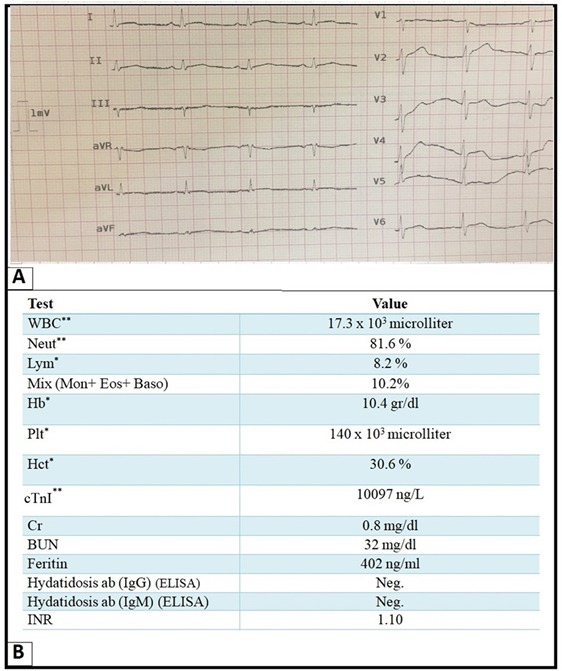
(A) Standard 12 lead ECG; Normal sinus rhythm with low voltage in all limb leads. (B) hematological and biochemical analysis, ** high level from standard value, * low level.

In physical examination, blood pressure was 100/65 mm Hg with a pulse rate of 70 bpm. He was afebrile. Heart sounds were muffle and there was no murmur. Electrocardiography showed a low QRS voltage in the limb leads (Figure 1A). Hematologic and biochemical laboratory exams showed leukocytosis, thrombocytopenia, anemia (normocytic, normochromic) and significant increase in cTnI level (Figure 1B).

**Figure 2:**
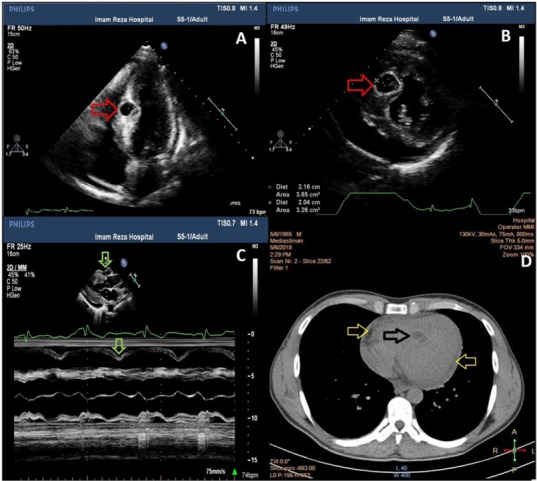
(A) Four chamber view (red arrow: well-defined cyst in IVS) B: Parasternal short axis view (red arrow: well-defined cyst in IVS with size: 2.0*2.1cm), C: Parasternal long axis view (M-mode); green arrows: RV diastolic collapse, D: HRCT of mediastinal window; black arrow: cyst-like mass in IVS, yellow arrows: pericardial effusion.

Transthoracic echocardiography (TTE) demonstrated a well-defined cyst (2×2.1 cm) with thickened wall in interventricular septum (IVS, at its mid-portion). Left ventricle (LV) was hypertrophied (septal thickness: 1.3 cm; posterior wall thickness: 1.44 cm) and its systolic function was reduced (LVEF #45%). Moreover, large pericardial effusion, with significant hemodynamic compromise (collapse of the right atrium (RA) and the RV) was evident (Figure 2 A, B & C). Pulmonary arterial pressure was normal.

High-resolution computed tomography (HRCT) revealed a hypodense, cystic like structure in IVS and also pericardial effusion (Figure 2D). HRCT of the lungs and CT of abdominal area showed no additional organ involvement. Because of echocardiographic evidence of tamponade, increase in cTni and reduced LV ejection fraction, we consider perimyocarditis which can be related to hydatid cyst reactively. Therefore, surgery was planned for pericardiocentesis and excisional biopsy of cytic-like mass.

**Figure 3:**
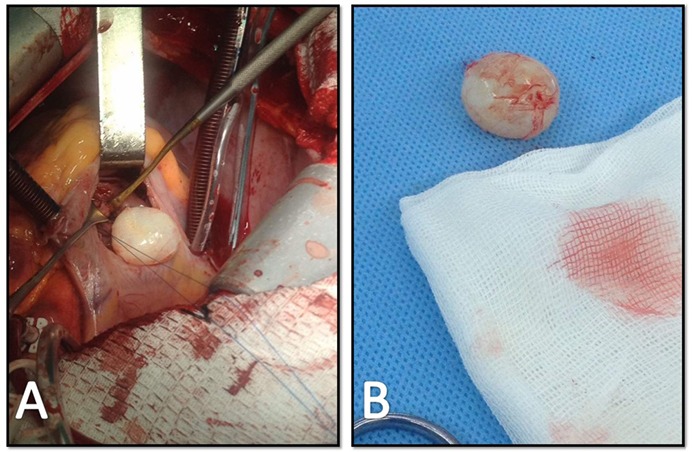
A. The view of the hydatid cyst in ventricular septum, B. Gross appearance of hydatid cyst (size: 2.0*2.1 cm)

Under general anesthesia, median sternotomy was undertaken. After cardiopulmonary bypass (CPB), cardioplegic solution infused into aortic root to introduce cardiac arrest. After right atrium opening, IVS mass was seen and total resection was performed without any damage or rupture of the cyst wall (Figure 3A & B).

**Figure 4:**
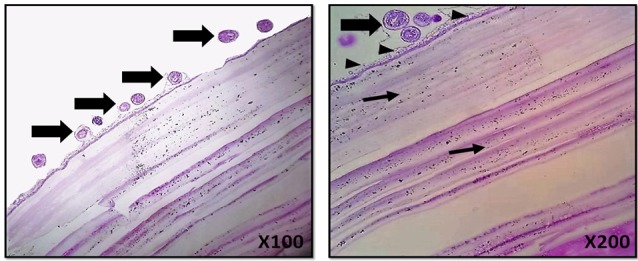
Micrographs of the cystic echinococcosis. Histopathological examination of the harvested specimen demonstrating the multilayer laminated cyst (H&E). Thick arrows: protoscolices, Thin arrows: laminated membrane, Arrowheads: germinal layer.

Micrographs of prepared histological slides from of the harvested sample (cyst) demonstrate a typical hydatid cyst. Histopathological evaluation showed a capsule including several protoscolices. The cyst consists of three layers: Outer, intermediate, and inner layers. The outer showed a connective tissue capsule, the intermediate layer showed non-nucleated, and several loosely arranged narrow laminations. The inner layer showed a germinal layer which was a nucleated layer and consisted of free fluid (Figure 4).

After histopathological report, the final diagnosis of a hydatidosis was established. Oral albendazole (400 mg/twice a day) was started for 4 weeks after final diagnosis.

## Discussion

Cardiac echinococcosis is a rare condition and the symptoms vary from case to case depending upon the size and site of infection.^6^ The development of hydatidosis is usually asymptomatic and only 10% of patients with cardiac infection are symptomatic.^7^ Because of life threatening complications of hydatid cyst such as compression, rupture or stenosis of vital structures and etc, surgical treatment is a necessity.^8^

Left ventricle is the most common area which involved with the hydatid cyst (55%-60%), because of its maximum blood supply.^9^ Therefore, one of the most important causes for ventricular aneurysm or cystic cardiac lesions is hydatid cyst, especially in endemic areas.^9, 10^ Interventricular septum is rarely involved in cardiac hydatid cyst (4%).^11^

Although continues contractions of heart provide a natural resistance for invasion of this parasite, this mechanism is not always effective and larva can infect the myocardial tissue in rare cases.^12^ After infection of heart, the cyst forms and grows between the myocardial fibers which may have no signs or symptoms. Clinical manifestations of disease are including precordial pain, dyspnea, obstruct the blood flow, embolism, and also invade the cardiac conduction system and cause arrhythmia or block.^12^ In some cases, rupture of cyst may cause anaphylactic shock or tamponade and coronary artery diseases.^13^

As described previously, echocardiography is a sensitive tool for diagnosis of cardiac masses including cysts or tumors. However, modern imaging techniques such as MRI or CT scan can be used to find the accurate location of lesion or additional information for involvement of other tissues.^14^ Hydatid cyst can be distinguished from other cyst-like lesions by calcification of cyst layers which may found in CT scan or presence of daughter cysts, and membrane detachment in MRI images.^14^ Like our case, negative results for serologic test of hydatidosis is not enough to role out this diagnosis and surgical treatment and subsequent histopathology results are necessary for final diagnosis. Moreover, in our case, hydatid cyst caused a perimyocarditis which considered as a rare presentation in cardiac echinococcosis.^12,13^

The treatment of choice for the most cases of cardiac echinococcosis is cardiac surgery; however this technique and the process of surgery differs from case to case.^13^ During the surgery, use of scolicidal solutions such as 1% iodine solution, ethanol 80%, NaCl 10%, chlorhexidine and methylene blue can reduce the risk of fluid leakage from hydatid cyst and extension into the adjacent tissues.^15^ After a successful surgical procedure, the duration of treatment by antiparasitic drugs and antibiotic agents is variable. Hopefully, our patient was successfully recovered after his cardiac surgery and treatment by albendazole for 4 weeks post-surgery. Patient follow-up at 1 and 4 weeks after discharge revealed that the general appearance of patient was good and echocardiographic results were normal with improved LV systolic function (LVEF# %50).

In endemic areas, hydatid cyst should be considered in differential diagnosis of any cardiac cystic-like lesions with heterogeneous echogenicity. A key component to successful therapy of cardiac hydatidosis depends on the early diagnosis of lesion and related surgical treatment.

## Competing interests

The authors declare that there is no conflict of interest.

## Ethical approval

Ethical approval for this study (No. 6717332) was provided by the Ethical Committee of Mashhad University of Medical Sciences, Mashhad, Iran on 29 January 2019. Also, the patient’s consent was obtained.

## Acknowledgments

The authors would like to thank Dr. Hossienikhah for his technical support during cardiac surgery.

## References

[1] Grosso G, Gruttadauria S, Biondi A, Marventano S, Mistretta A (2012). Worldwide epidemiology of liver hydatidosis including the Mediterranean area. World J Gastroenterol.

[2] Mihmanli M, Idiz UO, Kaya C, Demir U, Bostanci O, Omeroglu S (2016). Current status of diagnosis and treatment of hepatic echinococcosis. World J Hepatol.

[3] Mandal S, Mandal MD (2012). Human cystic echinococcosis: epidemiologic, zoonotic, clinical, diagnostic and therapeutic aspects. Asian Pac J Trop Med.

[4] Pakala T, Molina M, Wu GY (2016). Hepatic Echinococcal Cysts: A Review. J Clin Transl Hepatol.

[5] Dursun M, Terzibasioglu E, Yilmaz R, Cekrezi B, Olgar S, Nisli K (2008). Cardiac hydatid disease: CT and MRI findings. AJR Am J Roentgenol.

[6] Shojaei E, Yassin Z, Rezahosseini O (2016). Cardiac Hydatid Cyst: A Case Report. Iran J Public Health.

[7] Ipek G, Omeroglu SN, Goksedef D, Balkanay OO, Kanbur E, Engin E (2011). Large cardiac hydatid cyst in the interventricular septum. Tex Heart Inst J.

[8] Ibn Elhadj Z, Boukhris M, Kammoun I, Halima AB, Addad F, Kachboura S (2014). Cardiac hydatid cyst revealed by ventricular tachycardia. J Saudi Heart Assoc.

[9] Poorzand H, Teshnizi MA, Baghini VS, Gifani M, Gholoobi A, Zirak N (2014). Giant cardiac hydatid cyst with rare adhesions. Hellenic J Cardiol.

[10] Oraha AY, Faqe DA, Kadoura M, Kakamad FH, Yaldo FF, Aziz SQ (2018). Cardiac Hydatid cysts; presentation and management A case series. Ann Med Surg (Lond).

[11] Sabzi F, Faraji R (2014). Hydatid cyst of the interventricular septum causing complete heart block and postoperative ventricular septal defect. Indian J Crit Care Med.

[12] Abtahi F, Mahmoody Y (2009). Myocardial Hydatid Cyst. An Uncommon Complication of Echinococcal Infection.

[13] Yaliniz H, Tokcan A, Salih OK, Ulus T (2006). Surgical treatment of cardiac hydatid disease: A report of 7 cases. Tex Heart Inst J.

[14] Canpolat U, Yorgun H, Sunman H, Aytemir K (2011). Cardiac hydatid cyst mimicking left ventricular aneurysm and diagnosed by magnetic resonance imaging. Turk Kardiyol Dern Ars.

[15] Besir Y, Gucu A, Surer S, Rodoplu O, Melek M, Tetik O (2013). Giant cardiac hydatid cyst in the interventricular septum protruding to right ventricular epicardium. Indian Heart J.

